# Protein–Polyelectrolyte Complexes and Micellar Assemblies

**DOI:** 10.3390/polym11071097

**Published:** 2019-06-28

**Authors:** Shang Gao, Advait Holkar, Samanvaya Srivastava

**Affiliations:** Department of Chemical and Biomolecular Engineering, University of California, Los Angeles, Los Angeles, CA 90095, USA

**Keywords:** complexation, self-assembly, protein delivery

## Abstract

In this review, we highlight the recent progress in our understanding of the structure, properties and applications of protein–polyelectrolyte complexes in both bulk and micellar assemblies. Protein–polyelectrolyte complexes form the basis of the genetic code, enable facile protein purification, and have emerged as enterprising candidates for simulating protocellular environments and as efficient enzymatic bioreactors. Such complexes undergo self-assembly in bulk due to a combined influence of electrostatic interactions and entropy gains from counterion release. Diversifying the self-assembly by incorporation of block polyelectrolytes has further enabled fabrication of protein–polyelectrolyte complex micelles that are multifunctional carriers for therapeutic targeted delivery of proteins such as enzymes and antibodies. We discuss research efforts focused on the structure, properties and applications of protein–polyelectrolyte complexes in both bulk and micellar assemblies, along with the influences of amphoteric nature of proteins accompanying patchy distribution of charges leading to unique phenomena including multiple complexation windows and complexation on the wrong side of the isoelectric point.

## 1. Introduction

**Figure 1:**
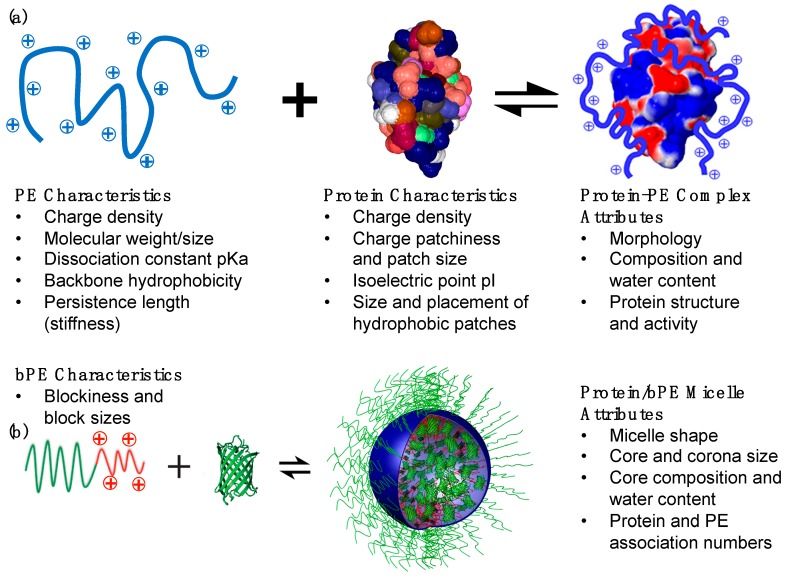
Schematics depicting complexation of proteins with (**a**) polyelectrolytes and (**b**) *block* polyelectrolytes to form protein–polyelectrolyte complexes and protein–*block* polyelectrolyte micelles, respectively. The key characteristics of the (*block*) polyelectrolytes and the proteins that regulate the properties of the complexes and the micelles are listed. The proteins depicted in the schematics are (**a**) chicken egg white lysozyme and (**b**) enhanced green fluorescent protein. The three-dimensional rendering of the lysozyme globules also depicts the diversity of the amino acid residues, colored differently, present on the globular surface. The rendering was produced using the RCSB PDB [34]; (**b**) was adapted with permission from Ref. [35]. Copyright 2015, American Chemical Society.

Mixtures of proteins and polyelectrolytes (PEs) in aqueous milieu interact and assemble together to form protein–polyelectrolyte (protein–PE) complexes [1,2,3,4,5]. The associative macromolecular phase separation results in recruitment of both the proteins and the PEs into the complex phase. The phenomena of complexation are driven by a combination of electrostatic interactions and entropy gains from the release of counterions confined in the vicinity of the protein globules and the PE chains [6,7,8,9]. In this regard, protein–PE complexes (Figure 1a) can be considered to belong to a large class of self-assemblies that form upon complexation of oppositely charged macroions in aqueous solutions, including polyelectrolyte complexes (PECs) [10], that form upon complexation between oppositely charged polyelectrolytes, and flocculation of oppositely charged polyelectrolytes and (nano)particles [11]. However, the amphoteric nature of the protein globules with patchy charge distribution on their surfaces differentiates them from uniformly charged macroions, polyelectrolytes or nanoparticles, and thus protein–PE complexes bear subtle yet substantial distinctions in their structures and properties from other electrostatic complex self-assemblies [12]. While the strong partitioning of proteins and the PEs in the complex phase results in high macromolecular concentrations in the complexes, the complexes still are water-rich phases and preserve the native environment for the protein globules which in most cases sustains or even enhances their activities.

Protein–PE complexes are found widely in natural and biological systems, with perhaps the most prominent example being that of chromosomes (deoxyribonucleic acid (DNA)-histone complexes) [13,14] that form the basis of life. Following the pioneering works of Morawetz and Hughes in 1952 in harnessing protein–PE complexation and subsequent precipitation of the complexes for protein fractionation [15], protein–PE complexes comprising synthetic polyelectrolytes have been explored in diverse technological settings [16,17]. Recent advances in polymer synthesis, biotechnology and characterization techniques coupled with the unique and advantageous attributes of protein–PE complexes have contributed immensely towards the widespread use of bulk phase separated protein–PE complexes in myriad biotechnological applications. These include protein separation, purification and concentration [3,18,19], protein stabilization [20,21,22,23], drug/protein delivery [24,25,26], synthetic bio-adhesives [27,28,29], tissue engineering [30], gene therapy [31] and encapsulants in the food industry [1,32,33].

The composition, structure and properties of the protein–PE complexes are all inherently dictated by the nature and strength of interactions of the protein globules and the PE chains with each other as well as with their surroundings [1,2,36,37,38]. These interactions, primarily electrostatic in nature, are influenced by a combination of density, distribution, patchiness and extent of ionization of the ionizable groups on the protein surface and on the PE chains. The charge state of PE chains can vary between fully ionized chains and fully deionized neutral chains and can be controlled by the pH of the solution. The degree of ionization of the PE chains can be quantized by using the Henderson–Hasselbalch equation and the dissociation constant pKa of the ionizable groups. Largely, polyacids and polybases can be considered to be fully ionized at pH > pKa + 1 and pH < pKa – 1, respectively. Proteins, in contrast, typically have both positively and negatively charged ionizable moieties on their surfaces. pH of the aqueous environment influences the extent of ionization of both kinds of charged moieties and consequently controls the net charge of the proteins. The pH value at which the proteins carry no net charge is referred to as its isoelectric point pI; proteins are positively charged at pH < pI and negatively charged at pH> pI. Lysozyme, for example, has an isoelectric point at pH = 11 and is therefore positively charged at pH = 7. The presence of salt ions can also influence the ionization of the charged moieties on both the PE chains and the proteins, influencing the strength of the electrostatic interactions between them. Thus, the structure and properties of protein–PE complexes can be finely tuned post-complexation externally by varying the ionic strength or pH of the solution [39,40].

In addition to the placement and extent of ionization of the charged groups, other PE and protein characteristics also influence the complex structure and properties. The size and the persistence length (stiffness) of the PE chains both influence the *compactness* of the complexes. Longer PE chains can bridge between protein globules, and floppier chains can conform to maximize adsorption on the protein surface, both leading to denser complexes [40,41,42,43]. The concentrations of the PE chains and the protein globules in the solution also determines the composition and the morphology of the complexes, with higher compositions leading to larger volumes of complexes and inducing morphology transitions from globular to mesh-like complexes [40]. Lastly, hydrophobic interactions between the PE backbone and the hydrophobic patches on the protein surface can, in some cases, reinforce and in other cases hinder complexation, and their roles need to be considered carefully when designing PEs for specific applications concerning protein–PE complexes [2,44,45]. The key characteristics of proteins and PEs and the tunable attributes of complexes are summarized in Figure 1a.

Conjugating the polyelectrolyte with a neutral hydrophilic polymer prevents bulk phase separation upon complexation of the *block* polyelectrolyte with proteins, leading to nanoscale colloidal assemblies with core–corona micellar architectures. These assemblies usually have a compact core comprising the proteins and the charged blocks surrounded by a dilute corona composed of the neutral blocks [46,47]. These micellar colloids, referred to in this article as protein/*b*PE micelles, can be considered to belong to a class of self-assemblies known as polyelectrolyte complex (PEC) micelles [46,47,48,49,50,51]. Self-assembly of PEC micelles was first demonstrated by Kataoka and coworkers in 1995 by mixing a pair of oppositely charged *block* copolymers poly(ethylene glycol)–*block*-poly(L-lysine) (PEG–*b*-P(Lys)] and poly(ethylene glycol)–*block*-poly(α,β-aspartic acid) [PEG–*b*-P(Asp)) [52]. Since then, extensive studies have been carried out on fundamental properties of PEC micelles as well as their applications as nanocarriers for delivery of biologically active charged macromolecules with therapeutic efficacies, including nucleic acids [53,54,55,56] and proteins/enzymes [57,58,59,60,61,62].

Various attributes of protein/*b*PE micelles, including their tunable nanoscale size (<100 nm), high protein loadings in the micelle cores, stable protein structure and targeted delivery have significantly expanded the scope of their use in therapeutic settings [24,25,26,48]. The characteristics of these colloidal micellar assemblies are governed by factors that affect bulk protein–PE complexation, as discussed earlier, as well as the characteristics of the neutral blocks, thus allowing for further tunability of micelle structure and properties. The overall size of the *b*PE and the relative sizes of the neutral and ionizable blocks both can be tuned to influence the size, shape and compositions of the micelles, with the core and the corona sizes, and the protein loading in the micelle cores all independently optimizable, as summarized in Figure 1b [53,58,63,64,65].

In this article, we review the recent research progress in the fundamental investigations and enterprising applications of protein–PE complexes as well as protein/*b*PE micellar assemblies. We begin with a short description of the unique nature of protein globules that differentiates them from other uniformly charged macroions in Section 2. Section 3 comprises a discussion on the thermodynamics, phase behavior, structure, properties and applications of bulk protein–PE complexes. Section 4 reviews the structure, properties and applications of protein/*b*PE micelles, followed by conclusions and current outlook in Section 5. While we primarily focus on bulk complexes and colloidal assemblies wherein proteins are major component of the systems, we also include discussions on inclusion of proteins as minor components in bulk polyelectrolyte complexes (Section 3.3.3) as well as PEC micelles (Section 4.2). Readers may refer to excellent reviews elsewhere for complexes of proteins with polyampholyte [66,67,68,69] and hetero-protein complexes [70,71]; these have not been discussed here to limit the scope of the review.

## 2. Proteins: Patchy Charged Nanoparticles or Amphoteric Polyelectrolytes?

**Figure 2:**
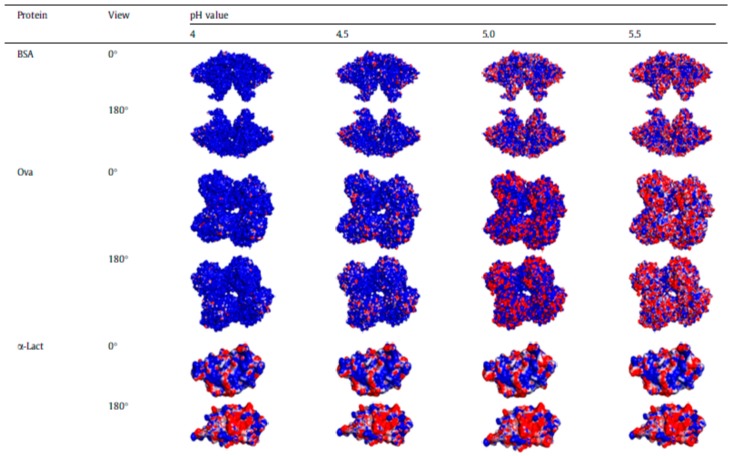
Simulations of surface charge distribution of bovine serum albumin (BSA), ovalbumin (Ova) and α-Lactoglobulin (α-Lact) at different pH with positively charged surfaces in blue, negatively charged surfaces in red, and neutral in white. Adapted with permission from Ref. [72]. Copyright 2015 Elsevier Ltd. All rights reserved.

Proteins are long chain polypeptides with particular sequences that fold into globular structures driven by secondary intermolecular interactions. The polypeptide chains contain both positively and negatively charged amino acids among other amino acids. Consequently, negatively and positively charged patches appear on the globular surfaces of proteins and enable complexation with oppositely charged *homo*- and *block* polyelectrolytes in aqueous media to form bulk and micellar (colloidal) complexes. Generally, proteins can be regarded as weakly charged nanoparticles with a low charge density and a charge sign that is dependent on pH. However, since the charges are not uniformly distributed, the approximation is crude at best. Figure 2 displays the surface structure of various proteins with varying pH, highlighting the evolution of surface charge patches. For instance, the net charges on a bovine serum albumin (BSA) globule at pH 4.5 is positive. However, the negatively charged patches (shown in red in the figure), when of appropriately high charge density and size, can still localize positively charged counterions in their vicinity. As discussed in the introduction, pH thus can be utilized as an essential tuning parameter to direct the complexation of proteins with PEs by altering the interaction strength between them.

The amphoteric nature of proteins is best highlighted in reports of complexation between proteins and PEs on the *wrong side of the protein isoelectric point* when the net protein charges and the PE charges are similar [73,74,75,76]. This behavior is typically ascribed to either patchiness of charges on the protein surface or charge regulation of proteins by the PE chains. The patchiness argument emphasizes the interactions and complexation between the PEs and the regions of proteins containing an excess of charge that is opposite to the net charge of the protein itself [74,76,77]. Sufficiently high charge density and large size of the oppositely charge patch can allow the polyelectrolyte chain to adsorb on the surface of the *multivalent counterion* charge patch while evading nearby similarly-charged patches, thus releasing counterions, increasing the entropy of the system and driving complexation [6,72,77]. Differences in charge distributions in proteins with similar pIs have shown significant differences in the pH at which they form complexes and as such have been used to formulate strategies for protein purification [18].

The charge regulation hypothesis, in contrast, states that the protein molecules and/or the PEs can adjust their overall charge state such that they acquire opposite charges and consequently complex [73]. Using self-consistent field theory, Biesheuvel et al. analyzed protein adsorption in polyelectrolyte brushes driven by charge regulation at a pH close to the protein pI where the brush and protein have the same charge [78,79], and hypothesized that proteins partitioning in the brush owing to the local negative charges in the brush might facilitate a switching of the net charge on the protein from negative to positive. Experimental studies, however, showed that adsorption of similarly-charged proteins to the brush occurred in the pH range farther from the pI than was theoretically predicted [78,79]. Generally, it can be concluded that both these mechanisms may be contributing towards the observations, with the relative contributions depending on the specific characteristics of the proteins and the PE chains.

## 3. Protein–Polyelectrolyte Bulk Complexes

Fundamental understanding of protein–PE interactions is crucial for understanding liquid–liquid phase separation leading to the formation of membraneless organelles inside cells (discussed further in Section 3.3.3) [80,81,82] as well as development of practical applications such as purification of therapeutic proteins (discussed further in Section 3.3.2) [3,72,78,83,84]. These motivations, among others, have led to a significant growth in research on protein–PE complexes in the past few decades.

### 3.1. Thermodynamics and Phase Behavior of Protein–Polyelectrolyte Complexes

Numerous studies have detailed the interactions and phase behavior of complexes comprising proteins and PEs and discussed the influence of ionic strength, PE characteristics (size, persistence length and charge density) and protein surface charge densities on the complexation phenomena [1,2,32,36,85]. Analogous to polyelectrolyte complexation, protein–PE complexation is generally accepted to be driven largely by a combination of electrostatic attraction between the constituents and entropy gains from counterion release. An excess of small ions can screen coulombic interactions as well as diminish the entropy gained from counterion release; thus, ionic strength of the solution significantly influences the compositions and volumes of the resulting phases.

#### 3.1.1. Phase Behavior

Phase behavior of protein–PE complexes is strongly dependent on the electrostatic interactions, which in turn are governed by several factors such as PE charge density, surface charge distribution of the protein and the solvent conditions (pH, ionic strength, dielectric constant). Additionally, the bulk concentrations of both the protein and the PE affect the composition and net charge of the microscopic complexes, thereby affecting the inter-complex interactions.

Various reports on the phase behaviors of protein–PE complexes have employed turbidimetry, notwithstanding the inherent empirical nature of the technique [2,86,87,88,89]. The highest turbidity is observed upon the aggregation and subsequent phase separation of the complexes. Thus, as expected, changes in turbidity occur on varying the pH of the solution as it influences both the protein surface charge and the PE degree of ionization, which in turn affect the complexation of the protein–PE pair as well as the aggregation of the complexes. Near-neutrality of the soluble complexes is required to induce aggregation and solution turbidity, and thus turbidimetry *only* reveals complexation windows wherein the complexes aggregate [86]. It should be noted that, in some special cases, aggregation of charged soluble complexes can be achieved due to the “inter-complex or intra-complex disproportionation” of charge as suggested by Zhang and Shklovskii [37,90].

**Figure 3:**
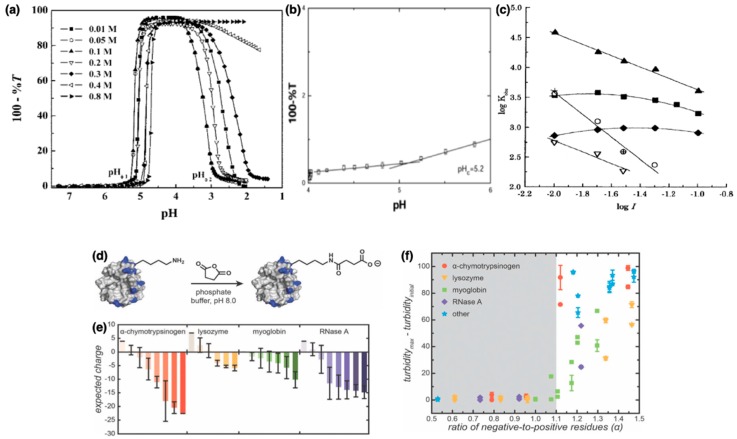
(**a**) turbidity of complexes of BLG with pectin with varying pH at different NaCl concentrations, where pH_ϕ1_ and pH_ϕ2_ denote pH at aggregation and dissolution of complexes. Reprinted with permission from Ref. [91]. Copyright 2007, American Chemical Society. (**b**) turbidity of BSA-PDADMAC as a function of pH at 50 mM salt, where pH_C_ denotes pH at which soluble complexes begin to form. Reprinted with permission from Ref. [92] Copyright 2010, American Chemical Society; (**c**) binding constants of BLG-NaPSS and BLG-PAMPS as a function of salt concentration: (Filled diamond) BLG-NaPSS at pH 7.0; (Filled square) BLG-NaPSS at pH 6.7; (Filled triangle) BLG-NaPSS at pH 6.3; (Open inverted triangle) BLG-PAMPS at pH 6.3; (Open Circle) BLG-PAMPS at pH 6.1; (Addition symbol) BLG-PAMPS at pH 6.1. Reprinted with permission from Ref. [76] Copyright 2003, American Chemical Society; (**d**) schematic for supercharging of proteins by succinic anhydride; (**e**) the expected charge on modified proteins with varying degrees of supercharging; (**f**) change in turbidity as a function of ratio of negative to positive charge residues on proteins. Grey area depicts complexes which do not phase separate; (**d**), (**e**) and (**f**) are reproduced from Ref. [93] with permission from the Royal Society of Chemistry.

Turbidity curves for proteins and anionic PEs with decreasing pH typically show first an increase in the turbidity at pH_ϕ1_ resulting from aggregation of complexes followed by a decrease in turbidity at pH_ϕ2_ denoting redissolution of complexes, as highlighted in Figure 3a for β-lactoglobulin (BLG)-pectin complexes [91]. Broad distribution of the PE molecular weights can result in the expansion of the range of pH over which aggregation occurs [86]. Another indicator of the onset of complexation corresponding to the formation of soluble complexes is the pH at which the slope of the turbidity curve departs from zero for the first time, denoted as pH_C_ (depicted in Figure 3b) [36]. pH_ϕ1_ and pH_ϕ2_ depend on the bulk stoichiometry as well as the molecular weight of the PE, all of which influence the long-range interactions between complexes. pH_C_ , however, has been argued to be influenced only by interactions prevalent on the length scales of protein sizes independent of the bulk concentrations and chain lengths [92].

Phase separated protein–PE complexes are classified either as coacervates or precipitates depending on their liquid-like or solid-like material response. Precipitation has been observed largely in cases involving strong polyions [1] and has been argued to be a kinetically controlled separation process [86,87], whereas coacervation is typically observed in protein–PE pairs with weaker interactions and results in reversible equilibrium phases [86]. The charge anisotropy of the proteins as well as the strength of the PE charges play significant roles in dictating the phase behavior [87]. In complexes comprising a weak polyanion and a protein wherein polyanion pK_a_ is lower but not too far from the protein pI, decreasing pH below pI leads to a concomitant increase in the positive charge on the protein and a decrease of polyanion ionization (as polyanion pK_a_ is approached), resulting in complexation and formation of coacervate droplets at pH < pH_ϕ1_. Continual decrease of pH continues to increase protein charge but reduces PE charge, eventually inhibiting complexation at pH < pH_ϕ2_ (see Figure 3a). For complexes comprising a strong polyanion and a protein wherein polyanion pK_a_ is far lower than the protein pI, decreasing the pH increases the protein charge while not substantively affecting the PE ionization, resulting in coacervation followed by precipitation [86].

Binding strengths of the protein–PE pairs and consequently their complexation is strongly influenced by addition of salt to the solution; increasing suppression of complexation with increasing salt concentrations is expected. In many cases, however, a maximum in the protein–PE binding strength is observed with increasing ionic strength. Figure 3c shows two such examples for complexes of BLG with sodium poly(styrene sulfonate) (NaPSS) and sodium poly(2-acrylamido-2-methylpropanesulfonate) (PAMPS), respectively [76]. This maximum is observed at ionic strengths at which the Debye length becomes similar to the protein radius and has been argued to result from decreasing inter-protein repulsion promoting complexation despite increasing screening of protein–PE interactions until an optimal ionic strength value. Corresponding minima in the pH_C_ and pH_ϕ1_ at the same ionic strength have also been observed with increasing ionic strengths of the solutions [92].

The complexation pH-windows have been further regulated by surface modification of proteins. Increase in protein charge density (supercharging) of model proteins (α-chymotrypsinogen, lysozyme, myoglobin, and RNase A) (Figure 3e) by reacting with succinic anhydride (Figure 3d) was observed to induce phase separation with polycations (quaternized poly(4-vinylpyridine) (qP_4_VP), quaternized poly(dimethylamino- ethylacrylate) (qPDMAEMA), and poly(oligoethyleneglycol- methacrylate-*block*-quaternized 4-vinylpyridine) (POEGMA-*b*-qP4VP)) when the ratio of negative to positive charge (α) was greater than 1.1-1.2, whereas phase separation did not occur without supercharging of proteins (Figure 3f) [93]. The pH for the onset of coacervation (pH_ϕ1_) showed a significant increase, the coacervate phase was more stable against dissolution on addition of salt, and higher protein fractions were observed in the coacervate phase on increasing α for all proteins. The secondary structures of the supercharged proteins, however, did not show significant differences from that of the unmodified proteins in most cases. In a similar vein, supercharged green fluorescent protein (GFP) with amino acid mutations showed phase separation with synthetic polyanions (poly(acrylic acid) (PAA) and poly(styrene sulfonate) (PSS)) and biological polyanions (DNA and ribonucleic acid (RNA)) [38]. Complexes comprising synthetic PEs showed a higher resistance to salt addition than those comprising biological PEs. Furthermore, complexation of relatively uniformly supercharged GFP was compared to the GFPs conjugated with ionic polypeptides at a single site, such that the net charged on the modified protein was the same [94]. Conjugated GFPs exclusively showed liquid–liquid phase separation, whereas some of the uniformly supercharged GFPs showed precipitation as well. In another study, the modification of hemoglobin by amidation of the carboxyl residues (aspartic and glutamic acid) was also reported to influence the phase behavior upon complexation with PAA; modified proteins formed soluble complexes with PAA while precipitation or macro-gel formation was reported for unmodified proteins [95].

Non-electrostatic interactions, namely hydrophobic interactions and hydrogen bonding also significantly affect complex structure and protein stability in the complexes. However, their influence on complexation has not been studied nearly as well as their electrostatic counterpart owing to the far more challenging systematic analyses [2]. Changes in the thermal transition temperature (as measured in dynamic scanning calorimetry experiments), indicating changes in protein structure, were observed for proteins chymotrypsin A, ribonuclease A, cytochrome C and lysozyme when complexed with polyanions poly(vinylsulphate) (PVS), heparin, PSS and Nafion [44]. Using a spectral probe aminoacridine to determine the nature of the protein–PE interactions, it was reported that while PVS and heparin interacted with proteins primarily via coulombic interactions, the relatively more hydrophobic PSS and Nafion had substantial non-electrostatic interactions with the proteins in addition to the electrostatic interactions. Correspondingly, larger structural perturbations of proteins were observed when they were complexed with PSS and Nafion as compared with PVS and heparin, indicating that hydrophobic interactions destabilized the protein far more as compared to electrostatic interactions. In another study, the rate of release of lysozyme from lysozyme–polyanion precipitates on dilution with salt solution was expectedly reported to reduce on increasing the PE charge density and on increasing PE chain lengths [45]. However, the release rates were observed to be lower from complexes comprising more hydrophobic polymethacrylates as compared to polyacrylate, indicating that non-coulombic interactions stabilized the complexes.

Enzyme-substrate specific interactions can also be affected by electrostatic complexation, thus influencing catalytic activity. For example, complexation between hyaluronic acid (HA) and bovine hyaluronidase (HAase) driven by non-specific electrostatic interactions leads to non-catalytic complexes [96]. Interestingly, addition of other proteins such as BSA and lysozyme that can compete with HAase to bind non-specifically with HA under suitable pH ranges leads to increased availability of HAase, thus resulting in an increase in the enzymatic activity of HAase upon addition of small amounts of the competing proteins.

#### 3.1.2. Thermodynamics of Complexation

An in-depth comprehension of the influences of protein and PE characteristics on the thermodynamics of complexation is imperative for the effective design and use of protein–PE complexes. Here, we summarize only the key features of thermodynamics of protein–PE complexation necessary for the completeness of the discussion; more details can be found in a recent review by Xu et al. [6].

**Figure 4:**
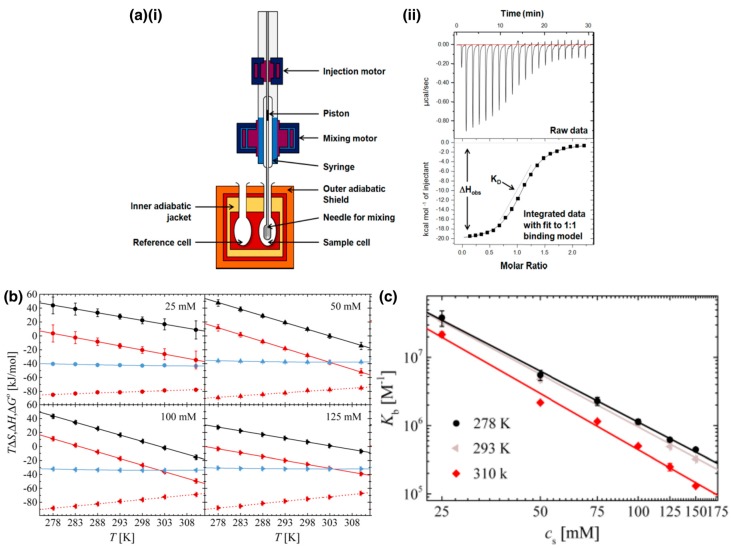
(**a**) (i) schematic of an isothermal titration calorimeter and (ii) results and analysis from a representative experiment. One reactant is injected at regular intervals of time into the sample cell holding the other reactant, and the energy required to maintain the sample cell at a constant temperature is measured as shown in (ii). The raw data are integrated and fit to a model to give the binding isotherm from which the binding enthalpy ΔH_obs_ and the binding affinity K_D_ are obtained. Reprinted with permission from Ref. [99]. Copyright 2015, American Chemical Society; (**b**) entropic (TΔS, black) and binding enthalpy (ΔH, red) contributions to total free energy ($\Delta G^{o}$, blue) at different salt concentration, along with the calorimetric enthalpy ($\Delta H_{ITC}$, dashed red) for the lysozyme- dendritic polyglycerol sulfate (dPGS) system. $\Delta H_{ITC}$ has contributions from enthalpy changes due ionization of buffer and of the ligand and changes in conformation; (**c**) dependence of binding constant (*K_b_*) on salt concentration for the lysozyme-dPGS system. (**b**) and (**c**) are adapted and reprinted with permission from Ref. [97]. Copyright 2018, American Chemical Society, respectively.

Isothermal titration calorimetry (ITC) is the most common technique for determining the thermodynamics of protein–PE binding during complexation. The titration data is integrated and is fit to a model (such as the single set of identical sites model) [97] from which the enthalpy, binding constant, free energy change and the entropy for the reaction is obtained (Figure 4a) [6,7,8,19,83,98,99]. The Gibbs free energy is expected to be negative for the spontaneous complexation of protein and PE. This is observed to be the case for binding of polyanion pectin and BLG at pH less than the pI of BLG [83]. Complexation on the *wrong side of the isoelectric point* is also of significant interest as it can be used not only to elucidate the roles of enthalpy and entropy but also of the protein surface charge. Complexation of PE and protein having the same net charge arises from binding between the PE and the oppositely charged patches on the protein and is driven by the release of condensed counterions; the enthalpy and entropy both increase during complexation [6,9,100,101], as shown in Figure 4b for dendritic polyglycerol sulfate (dPGS) and lysozyme complexes. The release of counterions in these cases has also been directly observed by SANS for lysozyme-PSS model system with deuterated tetramethylammonium counterions [102].

The change in free energy on complexation, $\Delta G_{r}$, depends on the charge density of the PE, surface charge density of the protein and the ionic strength. $\Delta G_{r}$ for complexation between an anionic linear PE and a protein at pH above the protein pI, with the protein and the PE both carrying net negative charges, can be estimated by [6,9]

(1) $$\frac{\Delta G_{r}}{kT}=\Delta N_{-}\ln\left[\frac{c_{s}}{c_{patch}}\right]+\Delta N_{+}\ln\left[\frac{c_{s}}{c_{PE}}\right],$$

where $\Delta N_{-}$ and $\Delta N_{+}$ is the number of negative and positive counterions released from the positive patch of the protein and from the PE, respectively, $c_{s}$ is the salt concentration in solution, $c_{patch}$ is the concentration of negative counterions on the positive charge patch of the protein and $c_{PE}$ is the concentration of the positive condensed counterions of the PE. This equation predicts a logarithmic dependence of free energy on salt concentration and is in agreement with the data obtained from the ITC experiments (Figure 4c) [6,103]. Similarly, the logarithm of the binding constant for BLG-PVS, BLG-PAMPS and BLG-PSS also showed a linear dependence on the logarithm of ionic strength when measured with frontal analysis continuous capillary electrophoresis [104]. It must be noted that deviations from linearity have been observed at lower salt concentrations when the repulsive interactions between the like charges of the protein and PE are no longer screened and contribute to the free energy, which are not considered in the equation provided above [6].

An important observation in numerous protein–PE systems is the enthalpy–entropy compensation (EEC) where changes in enthalpy and entropy at varying temperatures are such that the Gibbs free energy remains constant [97,99,105,106,107]. For instance, the ITC data for the dPGS and lysozyme at different salt concentrations shows that, even though the enthalpy or entropy change, the free energy remains constant as a function of temperature (Figure 4b) [97]. Complexation between a protein and a PE chain reduces the accessible states for two interacting components, leading to an overall decrease in the entropy of the two components. Stronger binding results in larger magnitudes of enthalpy changes but fewer accessible states (larger entropy changes), and vice versa, leading to EEC [99]. More importantly, this interdependent nature of entropy and enthalpy highlight the fastidiousness required to be exercised with measurements of the thermodynamic quantities [97,105].

### 3.2. Structure and Properties of Protein–Polyelectrolyte Complexes

**Figure 5:**
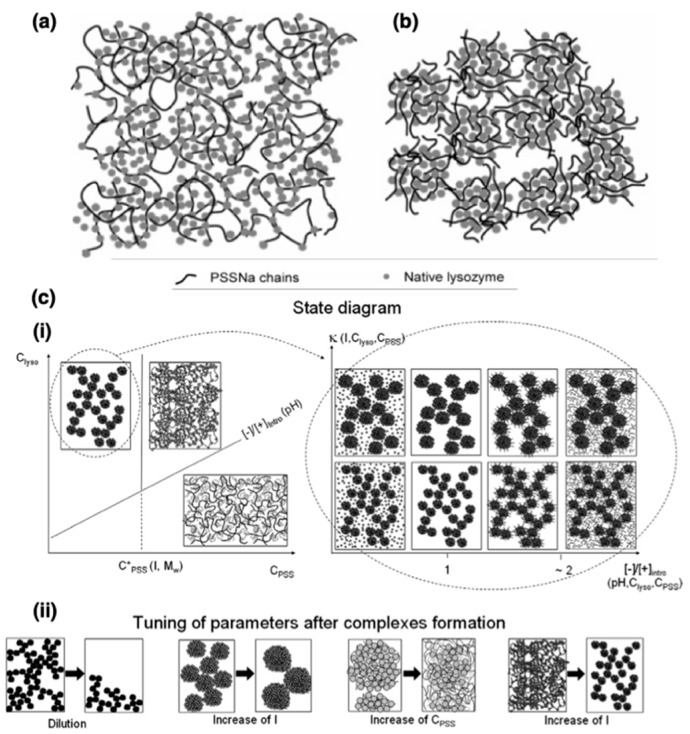
Schematics depicting structures of lysozyme-NaPSS complexes at PE:protein charge ratio of 3.33 with (**a**) long PE chains and (**b**) short PE chains. (**a**,**b**) are adapted with permission from Ref. [41]. Copyright 2005, American Chemical Society; **(c)** variation in structure of lysozyme-NaPSS complexes (**i**) in different regimes and (**ii**) on varying parameters post-complexation. Reprinted with permission from Ref. [40]. Copyright 2011 Elsevier Ltd. All rights reserved.

The structure of protein–PE complexes has been investigated using several techniques such as small angle neutron scattering (SANS) [39,41,42,43,108,109,110], dynamic light scattering (DLS) [43] and rheology [2,43]. Deuterium labelling of the PE chains has been employed to probe PE structure in lysozyme–NaPSS complexes using SANS measurements [41,111]. The scattering length densities (SLD) of unlabeled NaPSS and lysozyme are nearly equal. Therefore, in a solvent with a similar SLD, only the scattering from the deuterium labelled NaPSS (d-NaPSS) was detected, which allowed the lysozyme–NaPSS system to be studied in great detail. The conformation of individual NaPSS chains in the complex as well as the inter-chain interactions were quantified by varying the concentration of the deuterium labelled NaPSS chains [41]. Two regimes with distinct structures and macroscopic properties for the lysozyme-NaPSS complexes have been identified from SANS experiments. These constitute a semi-dilute gel-like regime with a mesh structure in which the lysozyme globules act as nodes interconnecting different PSS chains (Figure 5a) and a liquid-like dilute regime comprising densely packed spherical aggregates or globules of lysozyme and PSS chains (Figure 5b) [41]. A transition from the globular to the gel-like structure was observed upon increasing the PSS chain lengths from short chains (degree of polymerization *N* = 90) to long chains (*N* = 625) [41]. A similar transition was also observed with increasing NaPSS concentration (*N* = 800) at a constant ionic strength and protein concentration, or upon decreasing the ionic strength at constant NaPSS and protein concentration [42]. This transition from a globular to a gel-like structure was argued to be similar to the transition of a pure PE in solution from the dilute to the semi-dilute regime, which is observed at the critical concentration for chain overlap and is dependent on the size of the PE chains. The protein globules were envisaged as macroions that affect the electrostatic persistence length of PEs, leading to a decrease in chain dimensions and thereby increasing the critical overlap concentration. The extent to which the electrostatic persistence length and hence the critical overlap concentration of PE chains are affected depend on the strength of interaction of the protein–PE complex. It should be noted that the PE chain sizes may not be affected in case when protein–PE interactions are not significant [42]. Cousin et al. also reported the unfolding of lysozyme at large excess of NaPSS [41]. These results are summarized in Figure 5c(i) [40].

The critical overlap concentration of the PE can also be influenced by increasing ionic strength of the solution. Ionic strength of the solution can therefore be employed to tune the complex microstructure from gel-like to globular structures, with further addition of salt increasing the size of the complex globules (Figure 5c(ii)) [39,40]. Correspondingly, reducing the Debye length increased the size of the lysozyme-PSS complex globules [109]. The globules were observed to have a charge neutral core and either a positively or negatively charged shell, depending on whether the introduced charge ratio in the bulk ([−]/[+]_intro_) (the charge ratio introduced in the bulk system) has excess positive or excess negative charges, respectively (Figure 5c(i)). The complex globules were postulated to form via reaction limited colloidal aggregation, as deduced from their fractal dimension of ~2.1. In the complex globule regime, the addition of salt increased the size of the globules while shifting of [−]/[+]_intro_ to higher values by dilution with additional NaPSS did not alter the intra-globular structure but caused macroscopic destabilization of the globules (Figure 5c(ii)) [39,40].

SANS experiments on BSA-NaPSS complexes showed similar structures with a fractal network of globules (soluble complexes) at high pH and gel-like (mesh-like) structure at pH = 4 [108]. Interestingly, the structure of the complexes was reported to be independent of the protein–PE concentration ratios; this observation was justified by the argument that the excess of protein or PEs were not incorporated in the complexes and instead remain in the solution.

#### Effect of Polyelectrolyte Stiffness

Persistence length of the PE plays a dominant role in governing the structure of protein–PE complexes [112]. A comparison of neutron scattering spectra from lysozyme-pectin complexes with the earlier work [42] on lysozyme-NaPSS complexes revealed a trend of increasing chain flexibility resulting in higher water content in the complexes, thus leading to lower *compactness* of the complexes [112]. Flexible chains with small persistence lengths have access to more configurations allowing the complex to attain structures with lower free energies as compared to stiffer chains with larger persistence lengths. The structural changes due to variation in PE persistence length also lead to changes in properties such as rheology and diffusion modes in the complex [43]. Poly(diallyldimethylammonium chloride) (PDADMAC) and chitosan, of similar molecular weights and linear charge densities but differing in persistence lengths (2.5 nm and 6 nm for PDADMAC and chitosan, respectively), when complexing with BSA resulted in complexes with significantly different properties [43]. A 10-fold increase in scattering intensities at low wave vectors, corresponding to the presence of structures at length scales larger than 100 nm, was observed in SANS measurements in BSA-chitosan complexes as compared to BSA-PDADMAC systems. The pH of coacervation was also lower for the BSA-chitosan system. BSA-chitosan complexes showed a 10–50-fold drop in the viscosity on the increase of temperature from 12 °C to 25 °C, in contrast to the BSA-PDADMAC system, which didn't show any significant change. Similarly, the binding constant, signifying the interaction strength, for complexes of BLG with the PSS, PVS and PAMPS, as determined by frontal analysis continuous capillary electrophoresis, was reported to decrease in the same order [104]. The significantly smaller persistence length of PVS than of PAMPS can be attributed to the lower binding efficiency of PAMPS. However, PSS, which has a persistence length similar that of PVS, showed notably stronger binding with BLG than with PVS, possibly due to a higher charge density.

In a few special cases, increasing chain stiffness enabled the PEs to access more compact conformations around the protein globules, leading to counterintuitive trends of increasing complexation with increasing PE backbone stiffness. These effects were highlighted upon comparisons of binding affinities of various PEs with either BSA (with charge patches) or a homogenously charged micelle (dodecyltrimethylammonium bromide/Triton X-100 (DTAB/TX- 100)) [113]. Generally, reduction in chain stiffness leads to an increase in the binding between PEs and micelles as well as between PEs and proteins. However, in the case of a PAA and a 25%-*stat*-AMPS-75% acrylamide (AAm) copolyelectrolyte complexing with BSA, the latter, despite having lower charge density and lower flexibility, showed a higher affinity for BSA. The uncharged part of the copolyelectrolyte was proposed to arrange itself such as to reduce the repulsions with the positive “charge patch” of BSA by placing its uncharged part of the chain closer to the oppositely charged parts of BSA, leading to rearrangements that were unattainable by uniformly charged PAA chains.

### 3.3. Applications of Protein–Polyelectrolyte Complexes

**Figure 6:**
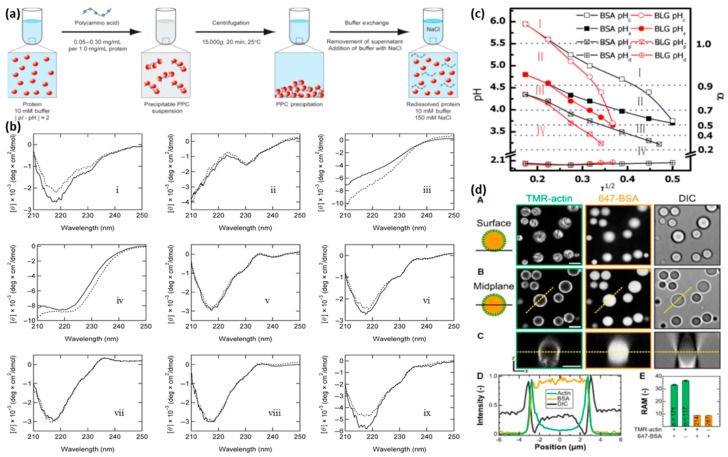
(**a**) schematic representation of concentration of proteins by precipitation and redissolution using poly(amino acid); (**b**) Far-UVCD spectra of i. panitumumab, ii. etanercept , iii. thyroglobulin, iv. L-asparaginase, v. adalimumab, vi. infliximab, vii. rituximab, viii. omalizumab and ix. IgG of native proteins (solid lines) and redissolved proteins (dotted lines) (**a**) and (**b**) are reprinted with permission from Ref. [114]. Copyright 2014 Wiley Periodicals, Inc. and the American Pharmacists Association; (**c**) phase boundaries of BSA and BLG with HA and the degree of ionization HA, α. pH_c_, pH_ϕ_, pH_p_ and pH_d_ are the pH values of soluble complex, phase transition, precipitation, and redissolution, respectively. Regions I−IV stand for noninteracting, soluble complex, coacervate, and precipitate, respectively. Adapted with permission from Ref. [19]. Copyright 2014, American Chemical Society; (**d**) (**A** and **B**) optical and confocal fluorescence micrographs showing PEC droplets (**right**) comprising both actin filaments (**left**) and BSA globules (**middle**). The focal planes are near the droplets-substrate interface (**A**) or near the midplane of the droplets (**B**). (**C**) An *x*–*z* cross section of the droplets evaluated from multiple images obtained at various z values. Scale bar, 5 μm in (A–**C**); (**D**) uniform distribution of BSA globules and peripheral enrichment of actin filaments in the PEC droplets as depicted from the normalized fluorescence intensity obtained from line scans along the dashed lines shown in (**B**) and (**C**); (**E**) average ratio of fluorescence intestines inside and outside the droplets, indicating a strong degree of partitioning of proteins in PEC droplets, in samples containing 0.5 mM actin, 0.5 mM BSA, or 0.25 mM actin and 0.25 mM BSA together. Reprinted with permission from Ref. [115]. Copyright 2018 Elsevier Ltd. All rights reserved.

Interactions in protein–PE complexes are understood broadly (largely electrostatic and entropically driven) and can be controlled by relatively straightforward methods (change in pH and ionic strength). Additionally, extensive research in the polymer synthesis has enabled preparation of PEs with a wide range of characteristics, enabling precise control of protein–PE interactions. These advances have paved the way for applications of these materials in diverse areas. An assortment of such applications, ranging from protein delivery to self-assembled artificial bioreactors, are highlighted in Figure 6.

#### 3.3.1. Protein Stability and Delivery

Polymers have been used for shielding proteins against surrounding environmental changes by forming protective shells around them [116,117,118,119,120]. This is especially important in therapeutic protein delivery where proteins may be exposed to proteolytic environments and degradation limits their uptake into cells. Encapsulation of proteins in polymer shells by covalent conjugation is shown to be effective in several instances [117,118,119,120]. Complexation of proteins with PEs has also be used for similar purposes. Use of protein–PE complexes for oral delivery of proteins has been explored extensively owing to the ability of the complexes to protect proteins against changes in pH and proteolytic degradation as well as overcoming the mucosal barrier [116].

High concentration of proteins can also be stored in protein–PE aggregate precipitates [22]. Kurinomaru et al. obtained high concentration of proteins (panitumumab, etanercept, thyroglobulin and others) in complexes by precipitation with cationic and anionic PEs (poly-L-lysine and poly-L-glutamic acid) followed by redissolution of the complexes in high salt environments (Figure 6a) without any significant changes in the activities of the proteins and antibodies [114]. The structures of nine redissolved proteins were analyzed using ultraviolet circular dichroism spectra (Figure 6b); only thyroglobulin (Figure 6b, panel iii) and L-asparaginase (Figure 6b, panel iv) were reported to exhibit minor peak shifts, indicating structural rearrangements [121]. Furthermore, precipitation has been proposed as an alternative to lyophilization as it provides thermal, physical and chemical (oxidation) stability, as seen in L-asparaginase-PE complexes [20]. Complexation and precipitation of proteins adalimumab and omalizumab with poly-L-glutamic acid reduced inactivation due to agitation and heat stresses, thus improving protein stability [21]. Similarly, heparin was shown to suppress and reverse aggregation of both BSA and antithrombin (AT) [23]. Even though heparin has a stronger affinity for AT than BSA due to the presence of a *more pronounced* positive patch, aggregation was inhibited more for BSA than AT, attributed to the higher aggregation of the uncomplexed monomers of AT than BSA.

#### 3.3.2. Protein Purification

Protein–PE complexation as a route for protein purification and isolation has garnered a significant amount of interest in recent years [3]. Selective complexation of BLG with the cationic PDADMAC was used for the purification BSA (pI ≈ 4.9) and BLG (pI ≈ 5.2) despite their similar pIs due to the larger negative charge patch on BLG as compared to BSA [18]. Selective complexation of BSA in a solution of BSA and BLG was also observed with anionic HA and a BSA yield of 90% purity was obtained for a 1:1 w/w mixture of the two [19]. Conditions for optimal yield were determined from the phase boundaries of soluble complex formation, coacervation, precipitation and redissolution of BSA and BLG with HA as depicted in Figure 6c. Similarly, purification of antibodies by complexation with PEs was used to reduce the number of purification steps by chromatography [84]. Yield by precipitation of monoclonal antibody by using a co-polymer of AMPS-*b*-4-(acryloylamino)benzoic acid (ABZ) at optimal conditions was 80% on average as compared to an average yield of 93% by affinity chromatography using protein A based purification. However, it was estimated based on material, labor and energy costs that the precipitation method was more cost effective for a large-scale production of antibodies. In addition, the structure of antibodies with and without precipitation showed no changes, implying that no protein deactivation had occurred. The impact of impurities in antibody purification based on the molecular weights, isoelectric points and surface charge of the impurities has also been studied [72]. Small impurities were found to interact with the complex but did not significantly change the precipitation of the antibody, whereas larger impurities reduced the yield of the antibody due to steric hindrance.

#### 3.3.3. Protein Encapsulation in Polyelectrolyte Complex Coacervates

Proteins can be incorporated in polyelectrolyte complexes (comprising two oppositely charged PEs) with high degrees of partitioning, thus leading to an effective *encapsulation* of the proteins in the complexes. Proteins are minor components in these three-component systems, and thus the inclusion of proteins typically doesn’t influence the properties of the bulk complexes. A discussion on the structure and properties of these systems was therefore not included in this review. However, it is pertinent to discuss these systems with respect to their prospective applications as vehicles for drug delivery and artificial bioreactors. Enzymes alcohol dehydrogenase and trypsin have been reported to retain their activities when complexed with PDADMAC [122]. The concentration of lysozyme *encapsulated* in coacervate droplets of PAA and poly-(N,N dimethylaminoethyl methacrylate) (PDMAEMA) was observed to be tunable by ionic strength and bulk concentration of lysozyme [123]. Poly(*L*-lysine) (PLK) and poly(*D*/*L*-glutamic acid) (PRE) coacervates were used to reversibly *encapsulate* BSA for application in drug delivery [124]. Coacervate droplets of PAA-polyallylamine hydrochloride (PAH) were also shown to stabilize the *encapsulated* protein against changes in pH and urea concentrations [125]. Furthermore, the secondary structure of the proteins was reported to be unchanged in both these studies. A systematic study of the *encapsulation* of BSA, lysozyme and hemoglobin in PLK and PRE revealed that large pH deviation from the protein pI led to greater uptake of proteins in the coacervate phase in all three cases, which was attributed to the increased charge on the protein [126]. On increasing the ionic strength of the solution, the partition coefficients of the proteins decreased. However, the efficiency of protein uptake in the coacervate phase went through a minimum in all three cases. It was argued that the increasing salt concentration affected not only the interaction of the proteins with the PEs but also the interaction between the PEs, thereby influencing the volume of the two phases [127]. The resultant changes in the coacervate volume contributed towards the non-monotonic trends of protein uptake efficiency with increasing salt concentrations. Furthermore, increasing the chain length from *n* = 50 to *n* = 800 showed a significant decrease of the partition coefficient only for lysozyme and hemoglobin, whereas no significant change was observed for BSA for the same. The efficiency of protein uptake in the coacervate decreased only for lysozyme on increasing chain size. Hence, while it may be possible to predict some qualitative trends derived from the appreciation of single PE and protein systems, a holistic understanding necessary for effective design of protein partitioning in coacervate systems is still lacking. In another study, *co-encapsulation* of BSA and globular actin was demonstrated in PLK-PRE coacervates (Figure 6d, panels A–C) with high degrees of partitioning for both the proteins (partition coefficients of ~10 and ~30, respectively; Figure 6d, panel E) [115]. Correspondingly, up to a 50-fold increase in the rate of actin filament assembly inside the droplets was reported and was attributed to a combination of high local actin concentrations, macromolecular crowding effects and changes in the local dielectric constants owing to high concentrations of PEs present in the droplets. Surprisingly, the assembled actin filaments were observed to concentrate on the periphery of the droplet (Figure 6d, panels A–C, column 1) as opposed to BSA which was uniformly dispersed (Figure 6d, panels A-C, column 2) in the droplets (Figure 6d, panel D).

## 4. Protein-Block Polyelectrolyte Complex Micelles

Protein drugs, including enzymes and antibodies, have become essential pharmaceutical products in recent years [128]. The development of protein therapeutics, however, has been hindered by the unstable nature of proteins, their degradation through proteolysis and denaturation, the immunogenicity of proteins (antibodies), and the lack of appropriate carriers for intracellular release. Encapsulation of proteins with poly(ethylene glycol) (PEG) through covalent linkage, known as PEGylation, has been widely used as a strategy to modulate protein pharmacokinetics, stability and immunogenicity. However, steric hindrance and chemical modification of the active sites may cause a decrease in protein activities upon PEGylation, and it may not be a feasible strategy for all proteins owing to a lack of appropriate chemical linkage sites [129]. Complexation with *block* polyelectrolytes has therefore emerged as a promising delivery methodology for therapeutic proteins.

Kataoka and coworkers have pioneered the synthesis and fabrication of supramolecular PEC micellar assemblies comprising oppositely charged macromolecules, with one component being a PEG-polyelectrolyte *block* polymer and the other being either synthetic or natural polyelectrolytes, including plasmid DNA, antisense oligonucleotides and charged proteins [52,53,54,57,130,131,132,133]. Proteins complex with the oppositely charged polyelectrolyte blocks that in turn act as wrapping materials for the proteins while the PEG blocks prevent macro-aggregation of the complexes, thus forming PEC micelles with sizes ranging in tens of nanometers and narrow size distributions. In contrast to PEC micelles comprising pairs of *block* copolymers, chain-length recognition [131] is not necessary for micellization of protein and PEG-*b*-PEs. The micelle core serves as a nano-reactor separated from the outer medium, wherein relatively small substrates could diffuse in or out and thus react with the entrapped proteins, and modulation of the targeted micelle properties, such as stability, solubility, and protein activity could be achieved by molecular engineering of the PEG-*b*-PE copolymers. Furthermore, proteins can be shielded from harsh conditions in the outer medium wherein loss of activity might occur, such as at high temperature or exposure to organic media. Consequently, these protein-containing structures have garnered interest both in laboratory and in vivo settings and have been employed for targeted, controlled delivery of therapeutic proteins as substitutes for PEGylation approaches [134,135].

### 4.1. Protein/Block Polyelectrolyte (bPE) Micelles

#### 4.1.1. Critical Association Concentrations and Micelle Structure and Properties

Egg white lysozyme has been extensively employed to investigate the structure of protein/*b*PE micelles owing to its well-understood structure and biological properties. Additionally, the isoelectric point of egg white lysozyme (~11) is sufficiently high to maintain a net positive charge on the protein surface across large pH ranges and thus enable micellization with anionic PEs. Harada et al. reported assembly of ~50 nm spherical micelles with narrow size distributions upon complexation of chicken egg white lysozyme and poly(ethylene glycol)-*b*-poly(aspartic acid) *b*PEs (PEG_275_-*b*-P(Asp)_15_) [57]. In contrast to precipitates that formed readily in the aqueous mixture of lysozymes and P(Asp) homopolymers, the micellar assemblies were stable against precipitation for months at room temperature. Static light scattering measurements indicated 36 lysozymes with 42 chains of PEG-*b*-P(Asp) packed in the micelles prepared at mixing ratio corresponding to matched macromolecular charges. Micelle sizes as deduced from dynamic light scattering measurements were reported to have no angular dependence, denoting that the micelles were spherical in shape, and the sizes were found to be independent of micelle concentrations, indicating no secondary aggregate formation [57].

**Figure 7:**
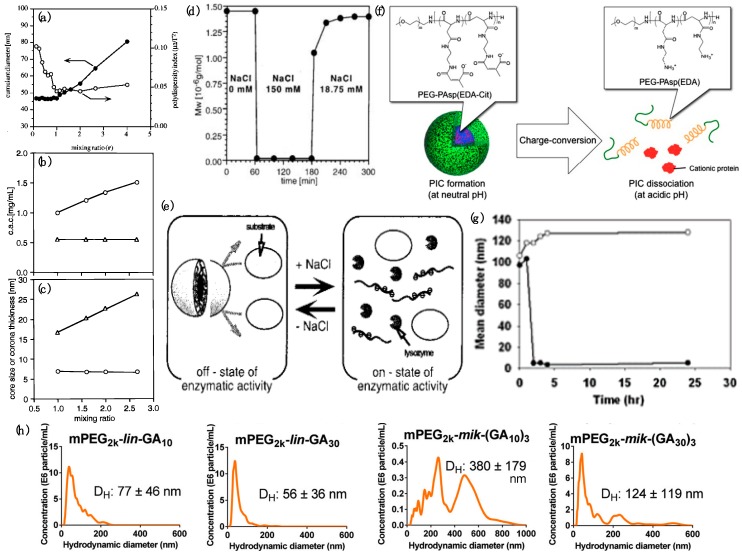
(**a**–**c**) various physical attributes of lysozyme/PEG-*b*-P(Asp) micelles varying with mixing ratio *r* at lysozyme concentration 2.0 mg/mL. (**a**) cumulant diameter (filled circles) and the polydispersity index (open circles); (**b**) critical association concentration (c.a.c.) (circles: c.a.c. of total concentration (lysozyme + P(Asp) + PEG); triangles: c.a.c. converted to the concentration of charged segments (lysozyme+P(Asp)); (**c**) core size (circles) and corona thickness (triangles) all changing with the mixing ratio *r*. (**a**) reprinted with permission from Ref. [58]. Copyright 1999 American Chemical Society. (**b**,**c**) adapted with permission from Ref. [57]. Copyright 1998 American Chemical Society; (**d**) fast and reversible dissociation and formation of protein–PE micelles upon exposure to high and low NaCl concentrations, respectively; (**e**) schematic illustration of the concomitant regulation of enzymatic activity of lysozymes upon incorporation and release from micelles; (**d**,**e**) adapted with permission from Ref. [59]. Copyright 1999 American Chemical Society; (**f**) schematic illustration of the stable micelle comprising PEG-*b*-pAsp(EDA-Cit) at neutral pH and the charge-conversion of *block* copolymer leading to micellar dissociation along with protein release at acidic pH. Reprinted with permission from Ref. [133]; (**g**) size of the protein/*b*PE micelles of PEG-*b*-pAsp(EDA-Cit) varying with time. Reprinted with permission from [136]. Copyright 2007 American Chemical Society; (**h**) micelle size distribution of mPEG_2k_-lin-GA_10_, mPEG_2k_-lin-GA_30_, mPEG_2k_-mik-(GA_10_)_3_ and mPEG_2k_-mik-(GA_30_)_3_-derived complexes with lysozyme at charge ratio 1.0. Reproduced from Ref. [137] by permission of the Royal Society of Chemistry.

Varying mixing ratios r of lysozyme and PEG–*b*-P(Asp) (*r* = [Asp in PEG-*b*–P(Asp)]/[Lys+Arg in lysozyme]) resulted in intriguing trends. For *r* < 1, the size and zeta potential of the micelles were reported independent of *r* (Figure 7a), indicating that the excess lysozyme present in the solution with *r* < 1 were not incorporated in the micelles [57]. For 1 < *r* < 2.67, the excess PEG-*b*–P(Asp) were incorporated in the micelles, leading to a near linear increase of the hydrodynamic size of the micelles with increasing *r* (Figure 7a) [58]. Correspondingly, the apparent critical association concentrations (CAC) of lysozyme and PEG-*b*–P(Asp) (total concentration of lysozyme + PEG-*b*-P(Asp)) for micellization increased from 1.0 mg/mL to 1.5 mg/mL (Figure 7b). However, the concentration of the charged lysozymes and charged P(Asp) segments in the micelle core were reported to be independent of *r* (0.55 mg/mL) (Figure 7b). Concomitantly, the core size was found to be independent of *r* at around 7 nm, while the corona thickness increased linearly with *r* (Figure 7c). Lysozyme was shown to have a nearly fixed association number ~50 in the micelle cores, whereas the number of PEG–*b*-P(Asp) chains in each micelle increased with increasing *r*. Thus, it was suggested that (i) the micelle core size was controlled by the length of charged P(Asp) blocks and (ii) the number of PEG-*b*–P(Asp) chains in the micelle dictate the thickness of the corona [58].

Completely reversible dissociation of lysozyme/PEG-*b*-P(Asp) micelles through exposure to ionic strength (NaCl concentration) jumps was also demonstrated as a handle to modulate the protein availability [59]. The molecular weight of the micelles was found to decrease from 1.5 × 10^6^ g/mol to 1.4 × 10^4^ g/mol upon exposure to 150 mM NaCl solutions, indicating complete dissociation of the micelles (Figure 7d). The micelles were found to reassemble upon addition of NaCl-free PBS, thus decreasing salt concentration to 18.75 mM, with their sizes stabilizing over 300 min, indicating complete reversibility formation of PEC micelles (Figure 7d). When encapsulated in the micelles, lysozymes displayed no cell lytic activities; upon release from the micelles at high NaCl concentrations the micelles recovered their cell lytic activities, thus enabling a facile *on–off control* of lysozyme activity (Figure 7e) [59]. Further control over the response of the micelle to external stimuli was attained by utilization of PE blocks exhibiting pH-responsive charge switching or conversion [136]. At pH 7.4, the citraconic amides in PEG-*b*-poly[(N’-citraconyl-2-aminoethyl)aspartamide] (PEG-*b*-pAsp(EDA-Cit) bear negative charges and thus readily complexed with cationic lysozyme to form micelles (Figure 7f). However, the citraconic amide groups degraded into cationic primary amines immediately when exposed to acidic pH = 5.5, resulting in repulsion between the protein and the PE and complete dissociation of the micelles within hours (Figure 7g), thus releasing the protein (Figure 7f) [136].

Similar modulation of the micelle assembly with pH jumps was reported for lysozyme/PEG-*b*-polymethacrylic acid (PMAA) micelles with complete dissociation at pH = 5 and reassembly at pH = 8 over many pH cycles [138]. Molecular dynamics simulation showed that the micellization between lysozyme and PEG-*b*-PMAA proceeded through a two-step mechanism; the first step comprised the electrostatically-driven adsorption of the charged side groups in the PMAA block onto the oppositely charged regions on the protein surface, and the second step consisted of compaction of the complex driven by attractive interactions between hydrophobic regions of lysozyme and the hydrophobic backbone of the PMAA [138]. More recently, complexation of lysozyme with sodium poly(sulfamate-carboxylate) isoprene-*block*-poly(ethylene oxide) (PSCI-*b*-PEO) anionic/neutral *block* copolymers was shown to result in micelle formation at neutral pH and in the salt concentration range of 0.05 M–0.15 M [139,140]. Micellar structure and stability were both shown to depend on the charge mixing ratio depend as well as on the composition of the *block* copolymer. The size and mass of the micelles were reported to decrease while the colloidal stability against 0.15 M salt concentration was reported to increase upon increasing the length of the neutral block in the copolymer [139]. Furthermore, lysozyme structure was shown to be slightly perturbed in the complexed phase and the cell-lytic activity was completely inhibited upon complexation, but both the structure and the protein activity made complete recovery upon micelle dissociation by exposure to 1M NaCl [140].

Electrophoresis and nanoparticle tracking analysis were employed to analyze the effect of PE architecture on complexation efficiency by comparing complexation of lysozyme with either linear or miktoarm star PEs. Linear polymer structures comprising PEG and polyglutamic acid (PGA) blocks were generally found to complex faster and more efficiently with the proteins as compared to miktoarm analogues with similar glutamic acid (GA) repeating units, as assessed from the narrowness of the size distribution of the self-assemblies. Additionally, longer PE blocks were found to complex more efficiently. Thus, the overall trends for complexation efficiency were summarized as PEG_2k_-*mik*-(PGA_10_)_3_ < PEG_2k_-*lin*-PGA_10_ < PEG_2k_-*mik*-(PGA_30_)_3_ < PEG_2k_-*lin*-PGA_30_ (Figure 7h) [137].

Graft copolymers were employed for entrapping proteins in an attempt to improve the tunability of core and corona sizes. Anionic graft copolymers poly(sodium acrylate-*co*-sodium 2-acrylamido-2-methyl-1-propanesulfonate)-*graft*-poly(*N,N*-dimethylacrylamide) (P(NaA-*co*-NaAMPS)-*g*-PDMAM) with neutral PDMAM blocks grafted onto polyelectrolyte backbone (P(NaA-*co*-NaAMPS) were shown to entrap BSA forming water-soluble micelles, albeit only when the fraction of the hydrophilic PDMAM side chains was more than 50% [141]. In another study, graft diblock polymer poly(ethylene glycol)-*graft*-poly(allyl amine) (PEG-*g*-PAA) was used to entrap *Aspergillus Niger Glucose oxidase* (GOD); the PAA backbone participated in complexation while the graft PEG chains formed the shell-structure. Mixtures with different mixing ratios formed transparent solutions and the formation of core–shell structure micelles was verified via DLS and SLS measurements indicating ~30 nm spheres. Additionally, upon entrapment, GOD were reported to retain their enzymatic activity [142].

Fluorescence correlation spectroscopy (FCS) in combination with DLS was employed as an alternate strategy to investigate the structure of enhanced green fluorescent protein (EGFP)/poly(2-methyl-vinyl-pyridinium)-*b*-PEO (P2MVP-*b*-PEO) micelles [35]. About 450 protein molecules were reported contained in each micelle with micelle size being 34 nm, and micellar structures were formed above 100 nM EGFP concentrations, irrespective of the polymer lengths. Interestingly, the positive charge fraction in the micelles cores was reported to be 0.65 instead of stoichiometric ratio of 0.5, indicating an excess of the PE charge in the micelles cores, and was attributed to a combination of induced charging of protein in the presence of the polycation and contribution of hydrophobic and other intermolecular interactions [143]. Fluorescence spectroscopy of co-micellized proteins mTurquoise2 and SYFP2 that exhibit Foerster resonance energy transfer (FRET) in protein/P2MVP-*b*-PEO micelles revealed a first-order kinetics for micelle formation as well as exchange of proteins between micelles [144]. The micelle formation pathway was argued to follow a two-state model with dynamic equilibrium between small near-neutral protein/*b*PE complexes and micelles. However, it was surprising that relatively short relaxation times of ~100s were reported for the micelle formation and exchange processes in conjunction with extremely stable micelles with the overall Δ*G* for the formation of a micelle estimated ~ −880 *k_B_T* [144].

#### 4.1.2. Influence of Micellization on Protein Structure and Activity

Cores of protein/*b*PE micelles can be envisaged as nano-compartments that isolate and protect the protein globules from the outer environment, shielded by a neutral, hydrophilic corona. Protein structures are generally understood to not alter upon micellization with *b*PEs. The secondary structure of lysozymes was shown to remain unchanged after micellization with the oppositely charged PEs [139,145], and similar unaltered structures were found in BSA [73] as well as α-amylase after binding with PEG-*b*-PAMA derivatives [146,147,148]. Compact cores of protein/PE micelles, however, in some cases can affect local structural changes of the proteins, which might influence their functionality and activity. Complexation with poly(ethylene glycol)-*b*-poly(L-aspartic acid) (mPEG-*b*-PLD) was shown to influence the secondary and tertiary structures lysozymes leading to partial unfolding of the protein. Interactions between the protein and the aspartic acid also inhibited protein’s reversible unfolding-refolding processes upon exposure to heating/cooling cycles [149]. Nolles et al. demonstrated deviations of the spectroscopic features of EGFP upon micellization with P2MVP-*b*-PEO [150]. Comparing the spectra between EGFP with its monomeric variant mEGFP (with suppressed dimerization) in and outside micelles revealed tight packings of GFP in the micelle cores resulting in its dimerization and subsequent changes in its absorption and excitation spectra. No such changes in the spectra were observed for micellized mEGFP. Furthermore, the spectra changes in EGFP vanished upon release of the protein from the micelles [150]. In a subsequent study, evolution of spectral properties of seven different fluorescent proteins derived from either *Aequorea victoria* GFP (*av*FPs) or from class Anthozoa (*an*FPs) were investigated upon micellization with P2MVP-*b*-PEO. Spectral properties of all *av*FPs that maintained monomeric structure in micelles were found to be largely insensitive to micellization, while *av*FPs were reported to suffer from large decreases in the fluorescence quantum yields owing to restricted dimerization upon micellization [151].

Protein activity against cellular substrates, as ascertained from the lytic activity of lysozyme against *Micrococcus luteus*, was found to correlate closely with the formation and dissociation of the lysozyme containing micelles, and thus could be influenced by external stimuli such as ionic strength [59] as well as the application of an electric field [61]. Steric repulsion by the corona restricted direct interactions of the entrapped lysozyme with large-size external substrates (*Micrococcus luteus* cell walls), which led to a complete inhibition of lytic activity upon complexation. Upon exposure to NaCl concentration of 150 mM for 300 s, the micelles dissociated and released lysozymes, and thus the lysis activity was readily recovered. Upon decreasing the NaCl concentrations, the micelles reassembled, leading to vanishing lytic activity of the proteins within minutes. This reversible regulation was demonstrated to be viable for multiple cycles without any loss of lysozyme lytic activity (Figure 7e) [59]. Similar pH- and salt-concentration responsive lytic activity regulation through reversible micellization of lysozymes within PEG-*b*-PMAA micelles were also demonstrated [138].

Lysozyme micelles were also employed to investigate protein activity in bioreactions involving small molecule substrates that could diffuse in and out of the micelles. Spectrophotometry was used to determine the rates of lysozyme catalyzed cleavage of *β*-1,4-glycosidic bonds in *p*-nitrophenyl-penta-*N*-acetyl-*β*-chitopentaoside, NP-(GlcNAc)_5_ [60], and reported enhancements in protein activities upon micellization. The activity of micellized lysozymes was found to increase by up to 100% in the micelles prepared at *r* = 2.667. Kinetic constants of enzymatic reaction were determined by using the double reciprocal plots as

(2) $$\frac{1}{\nu }=\frac{K_{m}}{V_{\max}}\frac{1}{\left[s\right]}+\frac{1}{V_{max}},$$

with $K_{m}$ being the Michaelis constant, $V_{\max}$ being the maximum velocity, ν being the initial rate and [s] being the concentration of the substrate. The maximum velocity ($V_{max}$) was found not to vary upon micellization, but Michaelis constant ($K_{m}$) decreased drastically. Partitioning and condensation of the substrate in the micelle corona was ascribed to the apparent decrease in $K_{m}$, which in turn allowed for modulation of protein activity through tuning of the corona thickness by varying the mixing ratio of PEG-P(Asp) to lysozyme (see Figure 7c) [60]. Upon decreasing the size of chains of *p*-nitro-phenyl-*N*-acetyl-*β*-chitooligosides (NAG) as substrate molecules ((NAG)_2_, (NAG)_3_, (NAG)_4_ and (NAG)_5_), the reaction rates were reported to increase by up to 100-fold for the smallest substrates ((NAG)_2_) as compared to only a two-fold increase for the largest substrates ((NAG)_5_) [61]. The unusual increase in lysozyme activity upon inclusion in the micelles was attributed to a combination of the condensation effect in the corona phase [60] and improved binding affinity of smaller substrates with the micellized proteins such that the Michaelis constant ($K_{m}$) was independent of size while the maximum velocity of the reaction increased with decreasing size of the substrate independent of micellization [61]. Interestingly, these improved binding affinities of the smaller substrates with lysozyme were demonstrated to be switchable by application of an electric field, with production of *p*-nitrophenol completely inhibited above a critical voltage. These observations were rationalized by the ability of external electric fields to influence the electrostatic interactions in the micelles and thus affect lysozyme activities [61].

**Figure 8:**
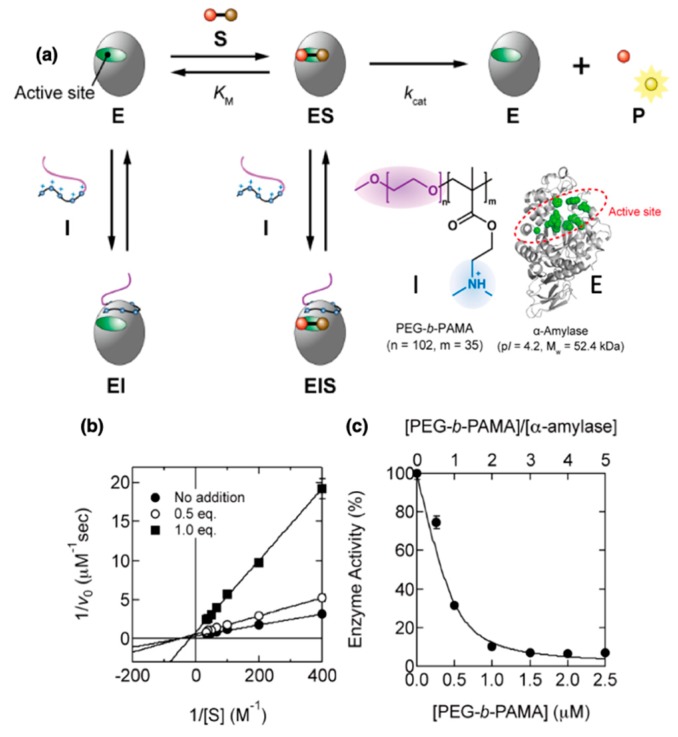
(**a**) schematic illustration of noncompetitive inhibition of α-amylase activity upon complexation of α-amylase with PEG-*b*-PAMA. The chemical structures of α-amylase (E; enzyme) and PEG-*b*-PAMA (I; inhibitor) are shown in the bottom right corner. The remaining symbols are described as follows: S: substrate; ES: enzyme−substrate complex; EI: enzyme−inhibitor complex; EIS: enzyme−inhibitor−substrate complex; P: product; (**b**) Lineweaver−Burk plots for substrate hydrolysis in 0.5 μM α-amylase solutions in MOPS buffer containing 0 (filled circles), 0.5 equivalents (open circles) and 1.0 equivalents (squares) of PEG-*b*-PAMA; (**c**) normalized activities of α-amylase in the presence of 20 mM substrate with various concentrations of PEG-*b*-PAMA. The experimental conditions were same for (**b**) and (**c**). Adapted with permission from Ref. [148]. Copyright 2017 American Chemical Society.

Specific interactions between the protein and the PE blocks can also influence the activity of the protein upon complexation and *entrapment* within the protein/*b*PE micelles. Complexation with PEG-*b*-P(Asp) was shown to accelerate the enzymatic activity of trypsin owing to the stabilization caused by hydrogen bonding between the imidazolium ion from the His residue in the catalytic site, the Asp-His-Ser triad, of trypsin with the Asp units in PEG-*b*-P(Asp) [65]. The extent of activity enhancement was found to be dependent on both size and concentration (relative to trypsin) of the PEG-P(Asp) chains. Furthermore, the amidase enzymatic reaction of trypsin accelerated upon micellization with poly(ethylene glycol)-*b*-poly(glutamic acid)) (PEG-*b*-PGA) and poly(ethylene glycol)-*b*-poly(methacrylic acid) (PEG-*b*-PMA) owing to formation of carboxylate/imidazolium pairs which enhanced the proton transfer process during the enzymatic reaction [152]. Conversely, complexation between cationic PEG-*b*-poly(N,N-dimethylaminoethyl)(PAMA) and anionic α-amylase [146,147,148] led to bonding of the *b*PE on the α-amylase surface, leading to noncompetitive inhibition of enzyme activity (Figure 8a). The noncompetitive binding of *b*PE on the enzyme surface was confirmed through computer simulations. $K_{m}$, estimated from Lineweaver–Burk plots (Figure 8b), for substrate hydrolysis by α-amylase was found to increase with increasing PEG-*b*-PAMA concentrations [148], corresponding to weakening of the affinity between the enzyme and the substrate in the presence of noncompetitive *b*PE binding with the protein, leading to decreasing enzyme activity (Figure 8c).

#### 4.1.3. Strategies for Stabilizing Protein/bPE Micelles

The utility of the protein/*b*PE micelles for in vivo application has further been modulated by tuning the robustness and the stability of protein/*b*PE micelles against variations in the ionic strength and pH of the environment [130,153]. Dissociation of the PEC micelles at low ionic strengths and at pH close to protein pI or PE pK_a_ can be alleviated while maintaining the durability and activity of entrapped proteins by harnessing additional stabilization resulting from hydrophobic interaction and chemical crosslinking. Jaturanpinyo et al. reported stabilization of trypsin/PEG–*b*-P(Asp) micelles by introducing glutaraldehyde as a crosslinker for the core. The aldehyde groups on glutaraldehyde and the primary amine groups on trypsin form a Schiff base, thus improving core stability in high ionic strength solutions [62]. The average diameter (70 nm–90 nm) of adequately crosslinked micelles stayed constant in high relative glutaraldehyde concentrations (GR > 10), GR defined as the total number of aldehyde groups in the glutaraldehyde solution versus the total number of Lys residues in trypsin, and high salt concentrations (NaCl 0-0.6 M). It must be noted that at GR values below 4, micelles were still destabilized upon exposure to salt beyond 0.15 M owing to incomplete crosslinking between trypsin and the primary amino group. Both free and micellized trypsin naturally denatured within a week, while core-stabilized micellized trypsin retained its enzymatic activity for >3 weeks. Analogously, stabilized nanosized protein/*b*PE micelles comprising anti-oxidant enzymes (superoxide dismutase (SOD1) or catalase) and oppositely charged *b*PEs were prepared by electrostatic self-assembly followed by covalent cross-linking (using glutaraldehyde, bis-(sulfosuccinimidyl)suberate sodium salt or 1-Ethyl-3-[3-dimethylaminopropyl]carbodiimide hydrochloride with *N*-hydroxysulfosuccinimide) to further immobilize protein containing micelles. Cross-linked micelles were demonstrated to exhibit increased stability in the blood stream as well as in the brain, and increased accumulation in the central nervous system when compared to uncrosslinked micelles and free SOD1 [24]. This approach of using glutaraldehyde crosslinkers, though, is not very feasible for biomedical applications owing to glutaraldehyde toxicity and irreversible crosslinking.

Micelle stability was also improved by harnessing cooperativity of molecular interactions by introducing hydrophobic interactions in conjunction with electrostatic interaction. Introduction of hydrophobic groups, phenyl (Phe), naphthyl (Nap), and pyrenyl (Py), to the ω-end of PEG-*b*-P(Asp) increased the association between lysozymes and PEG-*b*-P(Asp), leading to significantly lower critical association concentrations and improved stabilities in high ionic strength environments, albeit with deviations from the spherical shape of the micelles [154]. Micelles comprising PEG-*b*-P(Asp) with Py groups were reported to exhibit smallest variations in size upon exposure to NaCl concentrations up to 0.1 M; micelles with unfunctionalized PEG-*b*-P(Asp) dissociated at ~0.05M NaCl. Similarly, quaternization of the amine groups in PEG-*b*-PAMA polymers improved the stability of the α-amylase/PEG-*b*-PAMA micelles in 50 mM NaCl solutions [147].

In an innovative approach, enhanced micelle stability as well as efficient endosomal release was achieved through pH-responsive reversible chemical modifications of proteins, leading to charge reversal and higher charge density of the protein [155,156]. Lysine moieties on equine heart cytochrome C (CytC) was modified using citraconic anhydride and cis-aconitic anhydride to citraconic amide and cis-aconitic amide moieties, resulting in anionic Cyt–Cit and Cyt–Aco proteins that readily complexed with PEG–*b*-poly[N-{N’-(2-amino-ethyl)-2-aminoethyl}aspartamide] (PEG-*b*-pAsp(DET)) that were stable at physiological ionic strengths. However, upon exposure to pH 5.5 environment, the citraconic amide and cis-aconitic amide moieties degraded back to the primary amines, resulting in charge reversal of the proteins and rapid dissociation of the micelles [155]. The enhanced stability of the charge-conversional protein/*b*PE micelles led to extended circulation times of the micelles as well as controlled release of the proteins at endosomal pH, making them interesting candidates for in vivo protein delivery.

### 4.2. Protein Containing PEC Micelles

Diluting the core of micelles with homo- or *block* polyelectrolytes that are similarly charged to the net charge of the proteins enables further modulation of protein loading in the micellar cores and other micelle properties [157]. A significant portion of the work that has appeared in the recent literature on protein containing PEC micelles can be attributed to Lindhoud, Cohen Stuart and coworkers. Here, we briefly summarize their work on the structure, stability and response to changes in the ionic strength of the solutions.

#### Phase Behavior and Structure

**Figure 9:**
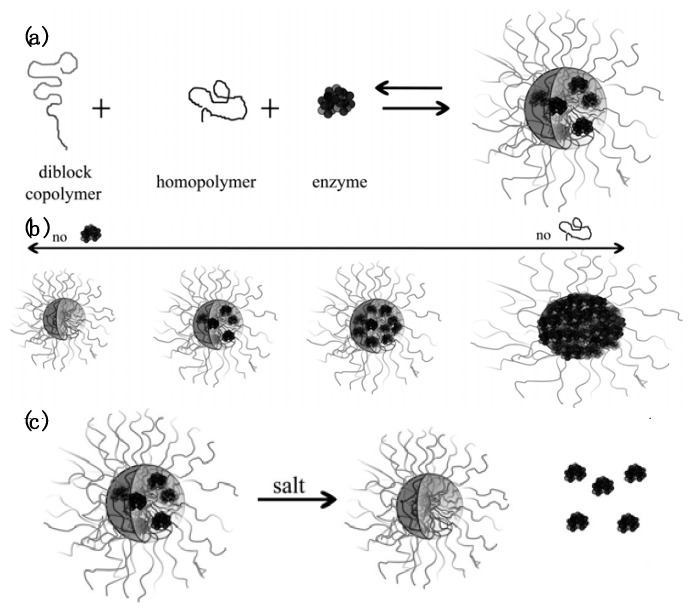
Schematics depicting (**a**) PEC micelle formation upon mixing of a charged diblock copolymer, an oppositely charged homopolymer and enzyme; (**b**) evolution of micelle morphology upon varying fraction of homopolymer and enzymes, and (**c**) release of enzyme from PEC micelles upon exposure to an external salt-jump stimulus. Reprinted with permission from Ref. [158]. Dissertation No. 4672, Library of Wageningen University.

The influence of adding a homopolymer, bearing a similar charge to the net charge on the proteins, to the protein/*b*PE micelle solution was systematically investigated using light scattering measurements [157,158]. Adding homopolymer PDMAEMA_150_ to the lysozyme/PAA_42_-*b*-PAAm_417_ micelles led to a stabilization of the micelles, with an increased resistance to salt, and could be attributed to the higher charge density of the homopolymer as compared to the protein globules (Figure 9a). The shape of the micelle cores also progressively evolved from an ellipsoid to a sphere with increasing fraction of homopolymer present in the core (Figure 9b), and correspondingly the loading of proteins in the core could be tuned by varying the fraction of homopolymer in the system [157]. Furthermore, it was argued that the order of mixing of the components could influence the micelle structure and properties as well their evolution over time [159]. Generally, it was suggested that an excess of homopolymer should be employed to form stable protein containing micelles [157,159].

The influence of salt on these protein containing micelles was also investigated for two representative systems: lysozyme containing micelles discussed previously (system *A*) [160] and lipase containing micelles composed of PAA_139_ and P2MVP_41_-*b*-PEO_205_ (system *B*) [161]. For similarly sized micelles (29 nm for system *A* vs. 23 nm for system *B*) at charge match conditions, the micelles were shown to contain 8, 29 and 2 molecules in system *A* and 40, 140 and 5 molecules in system *B* of homopolymer, diblock polymer and proteins, respectively [161]. In both cases, addition of salt led to a reduction of the size of the micellar cores, as estimated via SANS experiments, while the hydrodynamic radii, as estimated from DLS measurements, decreased and remained constant, respectively, for the two systems. The results suggested that, upon addition of salt, protein globules, owing to their lower charge densities than the homopolymers, were expelled from the cores of the micelles (Figure 9c). This expulsion led to a shrinkage of the micelle cores, and in lysozyme containing micelles also of the overall size of the micelles owing to the significantly larger fraction of proteins in the overall micelle compositions. The activity of lipase was also shown to increase upon encapsulations in PAA_139_/P2MVP_41_-*b*-PEO_205_ micelles, which was attributed to a combination of electrostatic interactions stabilizing an activated state of lipase and an accumulation of substrates in the micelles [162].

### 4.3. Applications of Protein–Polyelectrolyte Micelles

Polyelectrolyte complex based nanovehicles have emerged as versatile and robust platforms for delivering charged biomacromolecules, including nucleic acids [53,54,55,56] and proteins (enzymes) [57,58,59,60,61,62,163,164]. Incorporation of proteins in the protein-bPE micelles has several advantages: (i) the core of micelles provides a relatively water-rich environment, thus maintaining the protein molecules in their near-native states [165,166], (ii) shielding from the bulk solution by the neutral coronae mitigates immune responses, and (iii) high loading capacities implying that hundreds of protein globules can be incorporated in a single micelle, thus reducing the required dosage of micelles [35]. The water-rich core environment also allows for facile diffusion of small molecular substrates and cofactors in and out of the micelle cores, thus turning the micelles into nanoscale bioreactors.

**Figure 10:**
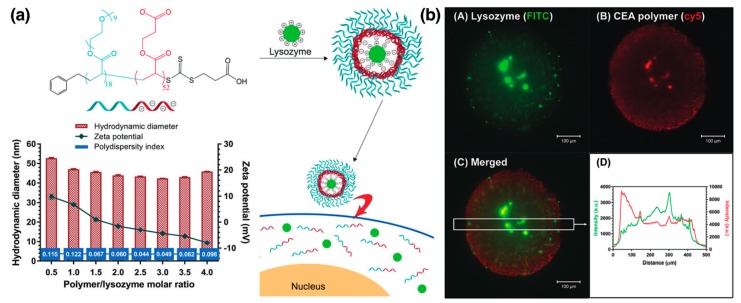
(**a**) schematic depicting the lysozyme/*b*PE micelle formation and the lysozyme cargos releasing into the cells. Also shown are the hydrodynamic sizes, zeta potentials and size polydispersity of the lysozyme/*b*PE micelles at various *b*PE/lysozyme ratios; (**b**) analysis of penetration ability of crosslinked lysozyme/*b*PE micelles in multicellular tumor spheroids using light-sheet microscopy. (A) FITC-labelled lysozyme (green), (B) Cy5-labelled CEA polymers (red), (C) Overlay of (A) and (B) showing respective distributions of polymers and lysozymes (D) Fluorescence intensity showing the relative distribution of polymer and lysozyme in the area marked with white box in (C). Adapted from Ref. [51] with permission from the Royal Society of Chemistry.

Stenzel and coworkers recently reported insightful experiments where confocal fluorescence light-sheet microscopy was employed to investigate in vitro transport processes and dissociation mechanisms of protein/*b*PE micelles resulting in the delivery of proteins in living cells (Figure 10a) [51]. Micelles comprising poly(ethylene glycol) methyl ether acrylate-*block*-poly-(2-carboxyethyl acrylate) (PEGMEA-*b*-PCEA) and hen egg white lysozyme with sizes ranging from 42 to 53 nm (Figure 10a) were employed. The two components of the micelles were fluorescently labelled separately with FITC labels on the proteins and Cy5 labels on the ionic segments of the *block* polyelectrolytes to facilitate imaging of individual components of the micelles upon transport and dissociation in multicellular tumor spheroids. The micelles were found to dissociate readily upon internalization within the spheroid upon interaction with other charged species inside the cytosol, and the *b*PEs were found only in outer layers of the 3D multicellular tumor spheroids. Lysozyme, conversely, were found to be transported in and out of the spheroid and were found to be distributed throughout the whole spheroid with a higher concentration at the periphery after 4 h incubation. Micelle stabilization by cross-linking of the core using 1,8-diaminooctane in the presence of *N*-(3-(dimethylamino)propyl)-*N’*-ethylcarbodiimide hydrochloride (EDC) and N-hydroxysuccinimide (NHS) leads to enhanced micelle stability and a relatively uniform delivery of proteins in the spheroid, as shown in Figure 10b [51].

**Figure 11:**
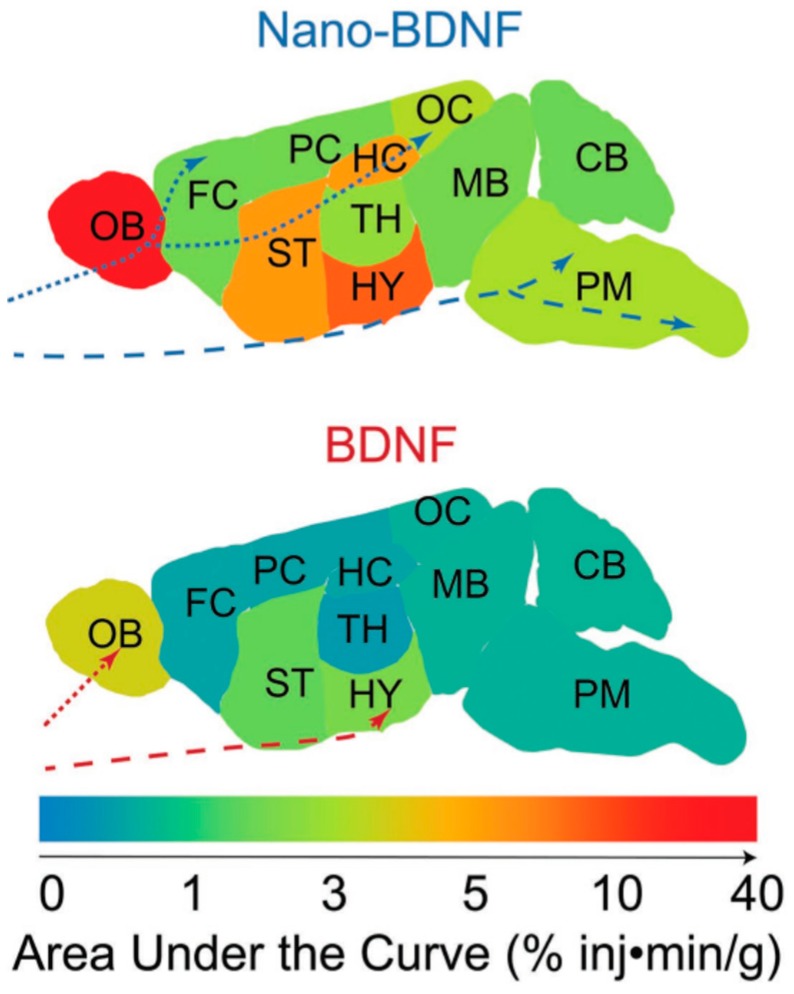
Heat maps depicting the detection of relative amount of brain-derived neutrophic factor, a neuroregenerative drug delivered either in native form (BDNF) or micellized with PEG-*b*-PLE (Nano-BDNF), in different regions of rodent brains over 30 min post-administration. The dashed and dotted lines correspond to the expected trajectories of Nano-BDNF and native BDNF via trigeminal and olfactory pathways, respectively. Reproduced with permission from Ref. [167].

Kabanov and coworkers demonstrated catalase/PEO-*g*-PEI micelles incorporated in a bone-marrow-derived macrophage system as a model for delivering catalase to Parkinson’s disease-affected regions in the brain [26]. At physiological pH and ionic strength, micelle-entrapped catalase was stable and retained antioxidant activity. Furthermore, in vivo studies demonstrated that significantly low enzyme degradation rates inside the host cells allowed for delivery of the catalase to targeted region in mouse brains. Subsequently, nanosized anti-oxidant enzymes/*b*PE crosslinked micelles with entrapped Cu/Zn superoxide dismutase (SOD1) or catalase enzymes, or co-entrapped SOD1 and catalase enzymes, were administered intravenously in mouse models and were found to have an increased stability in both blood and the brain as well as increased accumulation in the central nervous system [24]. The Cu/Zn superoxide dismutase (SOD1)/*b*PEs crosslinked micelles were also shown to consume reactive oxygen species radicals in two cell culture models in vitro, resulting in lower oxidative stresses, and were shown to heal ischemia/reperfusion-induced tissue injury in rat middle cerebral artery occlusion model along with improving sensorimotor functions in vivo upon intravenous administrations of the crosslinked micelles [25]. In another work, targeted delivery of brain-derived neutrophic factor (BDNF), a neuroregenerative drug encapsulated with PEG-*b*-PLE as micelles has been investigated [167]. The micelles were stable at physiological ionic strengths and in the presence of human serum albumin, serum immunoglobulin G and secretory immunoglobulin A which are commonly found in the body. The micelles also demonstrated selectivity in BDNF release from the micelle by its specific receptor, TrkB, and the activity of released BDNF remained unaltered. A 3.5 times increase in the overall accumulation of micellar BDNF in the brain was reported in mice as compared to native BDNF in a span of 30 min, with uniform distribution of BDNF in various parts of the brain achieved with micellar delivery (Figure 11). Micellar BDNF also resulted in significantly lower degradation of neurons as compared to that of native BDNF and saline. Moreover, the clearance rate of micellar BDNF from the brain was much slower (t_1/2_ = 167 min) than that for native BDNF (t_1/2_ = 25 min). The BDNF/PEG-*b*-PLS micellar system showcases a model system where the both stabilization of the drug as well as its target-specific delivery was demonstrated [167].

Avenues for targeted delivery have been explored by conjugating the neutral blocks of the *block* PEs with specific receptors thus decorating the micelle coronae with the receptors for targeted delivery. Micelles composed of poly(*L*-lysine)-*b*-poly(ethylene glycol) conjugated with folate (PLL-*b*-PEG-*b*-FOL) and negatively charged fluorescein isothiocyanate labelled BSA (FITC-BSA) were shown to target and bind to folate receptors which are typically overexpressed on various cancerous cells. Specifically, FITC-BSA/PLL-*b*-PEG-*b*-FOL micelles were shown to have an enhanced intracellular uptake in human epidermal carcinoma cells (over-expressing folate receptors) compared to FITC-BSA/PLL-*b*-PEG micelles or native FITC-BSA. [168].

**Figure 12:**
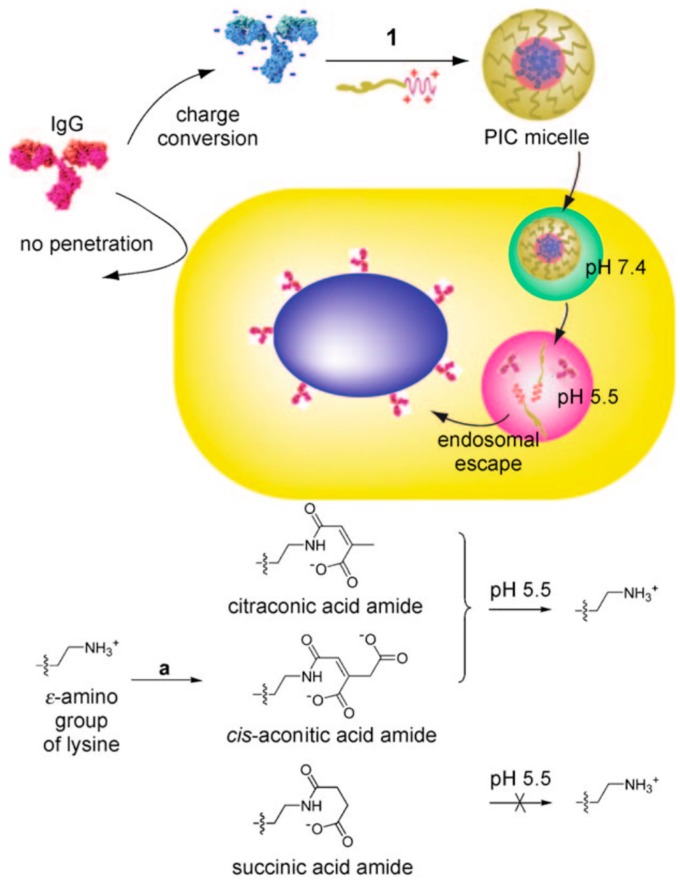
Schematic illustration of the use of protein/*b*PE micelles as a strategy for delivery of the protein. Charge-conversion of the protein is employed to gain precise pH-responsive control on micelle association and dissociation and delivery inside cells. Conjugation of IgG with citraconic anhydride, *cis*-aconitic anhydride, or succinic anhydride leads to conversion of free amines into an amide bond, which in stable and negatively charged at physiological pH. These charge-converted IgG globules can micellize with PEG-*b*-pAsp(DET) and be delivered to cells with minimal proteolytic degradation. In the two former cases, the amide bond can be cleaved upon exposure to endosomal pH, thus dissociating the micelle and releasing the IgG inside the cells. Reprinted with permission from Ref. [156].

Further improvements in the delivery efficiency of proteins using the protein/*b*PE micelles has been demonstrated by employing the pH-responsive charge-conversion method for the proteins as well as the *b*PEs. The cationic primary amines of lysine residues in IgG were converted to anionic sites by conjugation with citraconic anhydride [155,156,169]. The resultant citraconic acid amide was stable at neutral pH (7.4) but hydrolyzed at acidic pH of endosomes (pH ≈ 5.5). The citraconic anhydride-charged IgG formed stable protein/*b*PE micelles with PEG-*b*-cationic polyelectrolyte at pH=7.4. The micelles were readily internalized in the cells, and quickly dissociated at acidic pH (~5.5) in the endosomes upon charge reversal of the antibody, followed by endosomal release of the antibody into the cytoplasm for expressing the original antigen recognition (Figure 12).

A unique illustration of pH- and sugar-responsive protein containing PEC micelle was demonstrated using poly(ethylene glycol)-*b*-poly(glutamic acid-*co*-glutamicamidophenylboronic acid) and poly-(ethylene glycol)-*b*-poly(L-lysine-*co*-ε-3,4-dihydroxyphenylcarboxyl-L-lysine) [170]. These micelles were shown to (i) readily encapsulate charge proteins in their cores, irrespective of the sign of the charge, and (ii) disassemble and release their cargo when exposed to diols or acids. In vitro release studies confirmed micellar integrity at physiological conditions; the micelles disintegrated and protein cargo were released in the presence of excess fructose or at endosomal pH [170]. Furthermore, optimization of the micellar stability against swelling and dissociation in response to sugar or pH changes was demonstrated via modulation of cross-linking and hydrophobic interactions. These micelles were demonstrated to efficiently deliver cytochrome C to human liver cancer cell (HepG2) cells, thus improving cell apoptosis rates.

The activity of proteins (enzymes) entrapped into the PEC micelles has been thoroughly studied for the potential applications of enzyme-containing PEC micelles as nanoscale enzymatic reactors. The influence of micellization on protein activities has been discussed previously in the article. As the micellar cores form a segregated phase compartmentalized from the outer aqueous medium, which allow for entrapment and condensation of proteins as well as macromolecular crowding, they can serve as highly efficient nanoreactors for enzyme-catalyzed bioreactions [58,61,64,171,172].

## 5. Conclusions and Future Outlook

Protein–PE complexes and corresponding micellar assemblies are gaining popularity in biomedical and biotechnological realms. However, a greater understanding of the microscopic structure of these complexes is still needed to enable control over the prevailing interactions and production of complexes and self-assemblies with desired properties. Research efforts in the past have provided guidelines for understanding the role of electrostatic interactions and predicting the phase behaviour of complexes. However, the precise description of the complexation pathways still remain elusive, preventing a priori prediction of complex and micelle properties. Furthermore, the contributions of non-electrostatic interactions in complexation need detailed attention to establish structure–property relationships for these assemblies and ensure minimal variation in properties when processed at commercial scales. At the same time, investigations on the micellar interactions with biological systems, including micellar in vivo stability, biocompatibility, clearance pathways, and strategies for improvements on all these attributes are imperative to widen the scope of their use in diverse applications.
